# Harmonised global datasets of wind and solar farm locations and power

**DOI:** 10.1038/s41597-020-0469-8

**Published:** 2020-04-29

**Authors:** Sebastian Dunnett, Alessandro Sorichetta, Gail Taylor, Felix Eigenbrod

**Affiliations:** 1 School of Geography & Environmental Science, University of Southampton, Southampton, UK; 2 Biological Sciences, University of Southampton, Southampton, UK; 3 WorldPop, School of Geography & Environmental Science, University of Southampton, Southampton, UK; 4 Department of Plant Sciences, University of California, Davis, California USA

**Keywords:** Climate-change policy, Energy infrastructure, Wind energy, Energy access, Solar energy

## Abstract

Energy systems need decarbonisation in order to limit global warming to within safe limits. While global land planners are promising more of the planet’s limited space to wind and solar photovoltaic, there is little information on where current infrastructure is located. The majority of recent studies use land suitability for wind and solar, coupled with technical and socioeconomic constraints, as a proxy for actual location data. Here, we address this shortcoming. Using readily accessible OpenStreetMap data we present, to our knowledge, the first global, open-access, harmonised spatial datasets of wind and solar installations. We also include user friendly code to enable users to easily create newer versions of the dataset. Finally, we include first order estimates of power capacities of installations. We anticipate these data will be of widespread interest within global studies of the future potential and trade-offs associated with the global decarbonisation of energy systems.

## Abstract (summary)

**Table:** 

Measurement(s)	geographic location • power
Technology Type(s)	digital curation • computational modeling technique
Factor Type(s)	landscape area • panel area • turbines
Sample Characteristic - Environment	atmospheric wind • stellar radiation • power plant
Sample Characteristic - Location	Earth (planet)

Machine-accessible metadata file describing the reported data: 10.6084/m9.figshare.12063225

## Background & Summary

The estimated share of renewables in global electricity generation was more than 26% by the end of 2018^1^. Moreover, many national, regional and international policies mandate for ever larger renewable shares of electricity generation^2^.

Solar photovoltaic (PV) panels and wind turbines are by far the biggest drivers of the rapid increase in renewable energy electricity generation. Globally, in 2018, 100 gigawatts of solar PV were installed, contributing 55% of new renewable energy capacity; wind contributed the second largest share, with 28% of new renewable capacity^1^. Both technologies are well established and feature heavily in decarbonisation scenarios as proven concepts to generate emission-free electricity. Indeed, myriad studies present the goal of 100% renewable energy as eminently achievable with current technology^3^. For example, available wind power in Europe alone may be able to produce enough electricity for global demand to 2050, whilst replacing US hydroelectric dams with solar PV could produce equivalent power output on just 13% of the land^4,5^.

Despite this widespread interest in solar and wind, policy makers and governments have struggled to maintain robust geospatial information on the rapid expansion of renewable energy technologies. This lack of spatial data is problematic for several reasons. For example, the impacts of wind and solar installations on biodiversity are far from well known^6^, even at the local scale. The mortality effects of wind turbines on volant species are relatively well studied^7^, however ancillary effects such as noise, visual and landscape impacts are less well known. Furthermore, there may be significant trade-offs between increasing expansion of renewable energy globally, and efforts to reduce biodiversity decline through protected areas^8–10^. Another recent study suggests small-scale deployment of renewables has a lower impact on biodiversity than conventional energy, but the implications of scaling up renewable generation on biodiversity remain unknown^11^. Local-scale studies do show that siting of utility scale solar energy can have significant impacts on soil degradation and water availability^12^; and wind turbines can have significant effects on market prices^13^. However, hitherto studies have largely relied on the use of *suitability* maps for renewable energy which are not derived from historic placement of energy infrastructure but are rather based on purely climactic characteristics. As a result, they implicitly assume that the climactic characteristics will be the largest driver of installation placement^4,5,8,9,14–16^. At the global scale, a recent study used human influence as a proxy for where energy generation is occurring^11^. Both approaches are likely insufficient, as two UK-based studies showed that when location data are available, a variety of socioeconomic factors affect the siting of wind turbines and solar PV^17,18^. Sufficient location data would allow researchers to interrogate the socioeconomic drivers of renewable energy infrastructure siting at a global scale to produce probability surfaces for energy development^19^.

Despite the evident utility of location data, spatially explicit national data are only publicly available for a handful of countries^20–23^. Furthermore, often when they are available they are not open access. Global renewable energy data are readily available when spatially aggregated and summarised at the national scale (e.g. through the International Renewable Energy Agency - IRENA), but there is an urgent need for spatially explicit, global data describing the distribution of solar and wind installations. A harmonised spatial database could support data-driven indicators to track progress towards Sustainable Development Goals (SDGs)^24^, especially SDG 7 (Affordable and Clean Energy) and SDG 13 (Climate Action). A database would also allow the integration of global wind and solar installations with other geospatial datasets supporting SDGs, e.g. the World Database on Protected Areas informing the expansion of terrestrial protected areas for conserving threatened species supporting SDG 15 Life on Land.

Here, using OpenStreetMap infrastructure data, we present the first publicly available, spatially explicit, harmonised dataset describing global solar PV and wind turbine installations. These data are available in vector format, either as geopackages, shapefiles, or comma-delimited and describe groupings of wind turbines or solar PV, i.e. energy ‘farms’, as well as lone installations, i.e. a single wind turbine or solar panel. These data include metadata describing whether the location is urban or beside/on a water body, as well as an estimate of its power capacity, created using a predictive model detailed in this paper.

## Methods

### Data collection

#### OpenStreetMap structure

OpenStreetMap (OSM) is an open-source, collaborative global mapping project generated by a community of millions of users that can provide a unique insight into energy infrastructure locations. OSM data have an analogous structure to other geospatial data in that they describe the physical world with three different elements: points (e.g. street lamps, phone boxes), lines (e.g. roads, power lines), and polygons (e.g. parks, buildings). However, in OSM, point data are referred to as *nodes*, lines as *ways*, and polygons are described as *closed ways*. These georeferenced data are then given *tags* to ascribe the spatial data with meaning. Tags consist of two text fields, a *key* and a *value*. For example, a service road for a wind turbine or set of wind turbines could be a *way* tagged with key ‘highway’ and value ‘service’ to give a key/value pair *highway:service*. The value field provides more detail to the key classifier. More information on the structure of OSM data can be found on the OSM Wiki^25^.

#### OpenStreetMap key/value pair selection and extraction

To overcome the problem of inconsistent tagging in the OSM feature metadata, we conducted a preliminary analysis to determine the best key/value pairs to use as search terms for data extraction. We recorded the key/value pairs used for 50 randomly selected solar installations and 50 randomly selected wind installations with known locations. 50 random solar installations were selected from the Wiki-Solar dataset^26^, a dataset comprising 4129 solar projects. The wind installations were selected from a study into bird and bat mortality around wind turbines at 134 onshore sites^7^.

13 unique keys and 21 unique values were used to tag the solar sites in our test dataset (Table 1), with 5 unique keys and 11 unique values used to tag the wind sites (Table 2). The most common key/value pair for solar was *power* = *generator* paired with *generator:source* = *solar*. Frequently, OSM tags work in hierarchies; in this instance, features tagged *power* = *generator* should be further categorised to describe what *type* of energy is used for electricity generation. Moreover, anecdotally, it appears that the most common approach to tagging solar installations is to tag the entire area (*closed way* or polygon) as *power* = *plant*, while tagging groups of PV panels as *power* = *generator* and *generator:source* = *solar*.

**Table 1:** OpenStreetMap key/value pairs used for the sample 50 global solar installations.

Key	Values
barrier	fence; wall
building	yes
frequency	50
generator:method	photovoltaic
generator:output:electicity	10 MW; 3.5 MW; 5 MW; yes
generator:source	solar
generator:type	horizontal_axis; solar_photovoltaic_panel
highway	track
landuse	industrial
plant:output:electricity	250 MW; 290 MW; yes
plant:source	solar
power	generator; plant
wall	no

**Table 2:** OpenStreetMap key/value pairs used for the sample 50 global wind installations.

Key	Values
generator:method	wind_turbine
generator:output:electricity	1.75 MW; 2.1 MW; 2.3 MW; 3 MW; 800 kW; 900 kW; yes
generator:source	wind
generator:type	horizontal_axis
power	generator

For the sample of wind installations, tagging was much more straightforward. Again, the most common key/value pair to use was *power* = *generator*, this time coupled with *generator:source* = *wind*. However, there is one more use of *power* = *generator* without a corresponding *generator:source* tag within the sample of known wind installations. In reality, in most instances this sort of tagging omission would not be a problem with these data, as features generally occur together, and so any untagged elements are highly likely to be tagged by a different element within the same project.

Given that tag omissions were rare in the sample dataset, it was judged that using *generator:source* as a search key would capture most target features, with either *solar* or *wind* as the corresponding value. We therefore selected *generator:source* = *solar* and *generator:source* = *wind* as two of our search terms. We coupled these with *plant:source* = *solar* and *plant:source* = *wind* as the OSM Wiki suggested that the outer boundaries of renewable energy installations should be tagged thus.

Data were extracted using the R package *osmdata*^27^ to build queries to send to the Overpass API, a read-only API (available at overpass-turbo.eu) that allows for customised access to OSM data. For example, to search for solar PV OSM elements tagged as either *generator:source* = *solar* or *plant:source* = *solar* in a geographic area bound by bounding box *bbx*, a query can be built as follows:

$$\begin{array}{l}{\mathrm{query}}\,=\,{\mathrm{opq}}({\mathrm{bbox}}\,=\,{\mathrm{bbx}},{\mathrm{timeout}}\,=\,5000)\,\%\,> \, \% \\ \,{\mathrm{add}}\_{\mathrm{osm}}\_{\mathrm{feature}}(\mbox{``} {\mathrm{generator:source}}\mbox{''},\mbox{``} \mathrm{solar}\mbox{''})\, \% \, > \, \% \\ \,{\mathrm{add}}\_{\mathrm{osm}}\_{\mathrm{feature}}( \mbox{``}{\mathrm{plant:source}}\mbox{''}, \mbox{``}\mathrm{solar}\mbox{''})\end{array}$$

**Fig. 1:**
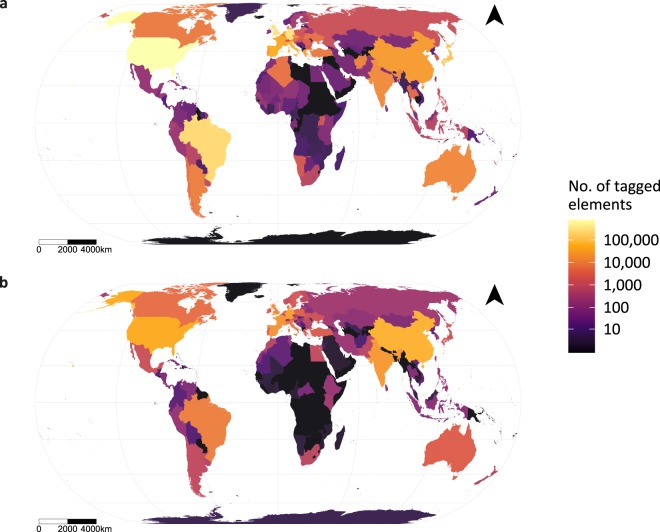
Numbers of OSM elements per country (point and polygon data) returned by search query for solar PV (**a**) and wind (**b**).

Applying this query globally resulted in four initial datasets: 326,234 solar polygons, 1,808,585 solar point data, 1,889 wind polygons, and 305,306 wind point data (Fig. 1).

### Data processing

#### Land cover

The first stage of data processing was to classify data located in areas identified as either water (both sea and inland bodies), or an urban centre. Offshore, rooftop and residential installations are very different in structure to field-scale solar and wind installations. Offshore turbines tend to be larger than onshore turbines to counteract high development costs^28^, while groups of residential buildings with single solar PV panels could conceivably be grouped as one solar installation, but do not act as one. Processing was done using the 2015 Global Human Settlement Layer (GHSL) in World Mollweide projection at 1 km resolution, reprojected to Eckert IV equal-area at 1 km resolution^29^. Urban areas were considered as any of the following municipal level categories in the GHSL dataset: City, Dense town, Semi-dense town and Suburbs. Water was taken straight from the GHSL categorisation.

#### Aggregating individual elements to installations

The raw OSM data extract contains sole polygons, sole points, as well as polygons and points within wider polygons. To counteract the potential for mis-tagging of these data as identified in our key/value pair analysis (Data Collection), we looked at the spatial clustering of the raw datasets to amalgamate any point and polygon data clearly referring to the same installation.

Firstly, we filtered any point already contained within a wider polygon, and the number of these points intersecting were recorded (strictly, the OSM guidance suggests tagging the wider installation as *power:plant* and *plants:source:* <source>, but in reality contributors tend to focus on the lowest unit: either a group of PV panels, or one wind turbine, tagging them with *power:generator* and *generator:source:* <source>). We then performed bespoke spatial clustering for each technology to group the remaining points that occur close together in space, as outlined below.

### Determining the scale for spatial clustering: spatial distribution of wind and solar features

In order to justify spatially clustering the remaining renewable energy point data into ‘farms’ based on their position in space relative to other points, we analysed the spatial characteristics of two large wind and solar databases, the United States Geological Survey (USGS) Wind Turbine Dataset (USWTD) and Wiki Solar^22,23,26^, to check whether they were significantly clustered in space.

We performed Ripley’s *K* function on both datasets^30^. Ripley’s *K* function returns a measure, *K*, for the spatial characteristics of a point pattern for a range of neighbourhood search radii, *r*. The function can be used to estimate whether a point pattern is clustered, dispersed, or distributed randomly in space. A theoretical *K* value, *K*(*r*)_theo_, is calculated based on a completely random Poisson point process at search radius *r*. If the observed *K* value at *r*, *K*(*r*)_obs_, is above *K*(*r*)_theo_, the point pattern is clustered at that spatial scale. If *K*(*r*)_obs_ is below *K*(*r*)_theo_, data are dispersed at that spatial scale. A difference of 0 suggests a completely random distribution.

In order to be calculated, the function requires a sensible study area. For the USWTD, this meant grouping the turbines by project (54481 turbines in 1311 projects). For Wiki Solar, we only had the point location of the installations. To address this, we applied circular buffers of the reported installation areas to the point locations and assumed all points from the OSM dataset in these areas belonged to the buffered project (63901 points in 1270 projects).

**Fig. 2:**
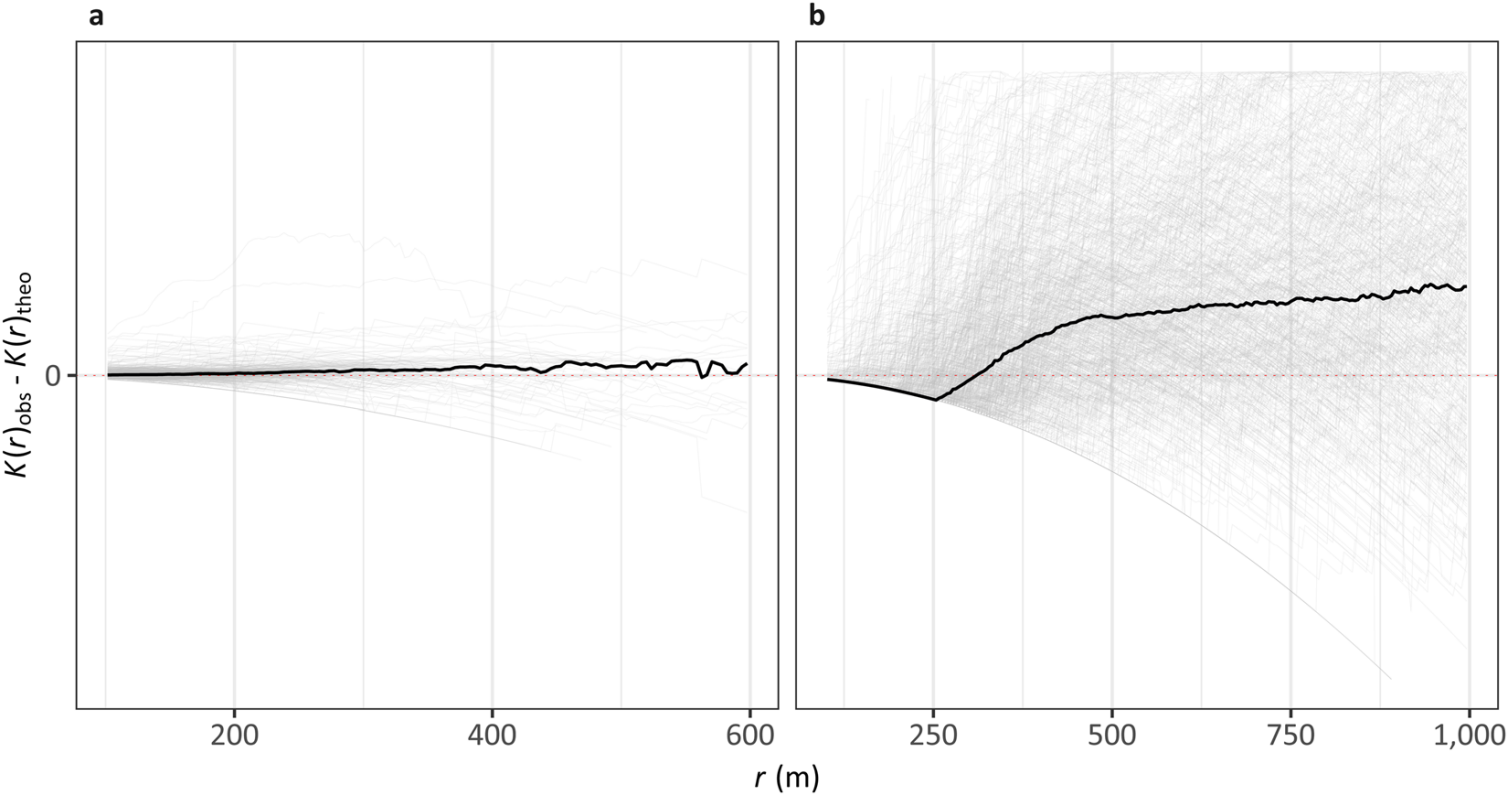
The difference between *K(r)*_*obs*_ and *K(r)*_*theo*_ per installation for the Wiki Solar dataset (**a**) and the USWTD (**b**) within different search radii, *r*. *K(r)*_*theo*_ represents the number of neighbours found within search distance *r* for a completely random Poisson point process. *K(r)*_*obs*_ represents the observed value of Ripley’s *K*. Where *K(r)*_*obs*_ > *K(r)*_*theo*_, data are clustered at that search radius; where *K(r)*_*obs*_ < *K(r)*_*theo*_, data are ordered. The horizontal dotted red line indicates a difference of 0, i.e. no different from a random process. Dark lines indicate the median differences of all projects.

Next, we applied Ripley’s *K* function to the point data in every project in both datasets. The result of this can be seen in Fig. 2. At shorter neighbourhood distances, we would expect our point patterns to be dispersed: wind turbines and solar panels require at least some dispersion from one another in order to work (e.g. to avoid wind wake for turbines, and shade for solar panels). For solar, the relationship is not clear, but for *r* values between 200 and 400 m, there appears to be a slight tendency towards clustering over dispersion (Fig. 2a). However, as *r* increases, we see a pronounced increase in clustering for wind; where 400 m < *r* < 1000 m, the majority of projects exhibit clustering over and above a random point process (Fig. 2b).

### Determining the neighbourhood radius for spatial clustering

Spatial clustering was achieved by running a density-based spatial clustering of applications with noise (DBSCAN) algorithm^31^. Given a set of points in space, DBSCAN groups together points that are closely packed together (points with many nearby neighbouring points), classifying as noise points that lie alone in low-density regions (whose nearest neighbours are too far away). The algorithm takes two parameters as arguments: ε, a neighbourhood search parameter, and *minPts*, the minimum number of points to form a cluster.

DBSCAN clustering is more appropriate than, for example, *k*-means clustering for these spatial data for two main reasons. Firstly, you do not need to specify the number of clusters *a priori*. As we are explicitly searching for the number of clusters in the data, this suits our needs. Secondly, DBSCAN can find arbitrarily shaped clusters. This is important as many wind farms are linear in shape and may be overlooked by more conventional clustering methods. In order to run this algorithm, two parameters need to be set: the neighbourhood radius (i.e. the search distance), ε, and *minPts*, the minimum number of points for the algorithm to consider a cluster. It is usually recommended that for setting parameters, *minPts* should be >2 so as to specifically look for *density*-based clusters. However, in order to extract linear clusters (which can occur for energy installations), *minPts* was set to 2. When *minPts* is set to 2, DBSCAN acts as a single-linkage hierarchical clustering algorithm truncated at ε. Whilst this avoids some of the pitfalls associated with DBSCAN of choosing an appropriate density^32^, single-linkage clustering is not without its disadvantages: this implementation can produce large clusters joined by one lone point. As a sensitivity analysis to this single-linkage effect, we repeated the analysis for *minPts* values of 3, 5 and 10 (Technical Validation). Values of 1 were not considered, as a lone wind turbine or solar panel cannot be considered a ‘farm’.

**Fig. 3:**
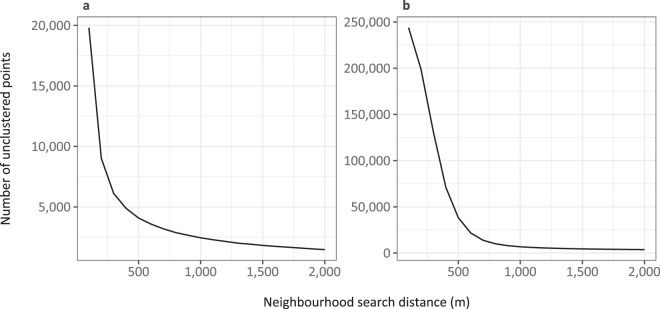
“Knee” plots for OSM solar (**a**) and wind (**b**) point data showing the number of unclustered points remaining for differing neighbourhood search radii. As the neighbourhood search distance increases, the number of unclustered points will eventually tend towards 0. Where the plot ‘turns’ indicates a sensible neighbourhood distance which captures the majority of points; for wind, this corroborates the previous analyses at ~800 m. For solar, it is more unclear but looks to lie in the range 300–500 m.

For wind, the neighbourhood radius (ε) has been discussed at length in the energy literature through the lens of the optimal spacing of wind turbines in order to maximise wind speed for each turbine. This varies widely but is largely considered to be in the range of 3–10 rotor diameters^33^. The median rotor diameter in the US and German turbine datasets were 87 and 71 m respectively, thus ε was set to 800 m. This also falls within the range 400–1000 m suggested by the Ripley’s K function analysis. Running DBSCAN on the OSM wind point data with differing values of ε suggests that 800 m is a sensible neighbourhood size (Fig. 3).

For the solar data, this proved much more difficult as there is no theoretical basis for spacing of panels in solar farms. There is recommended spacing at the very lowest level, as rows of panels are required to be a certain distance apart (dependent on their solar incidence slope) so as not to shade each other. However, we are interested in spatial clustering at the higher level of panel architecture, i.e. an array of multiple panel rows, on which there are no restrictions. The Ripley’s *K* function analysis suggested an optimal clustering distance of 200-400 m. Again, running DBSCAN with differing values of ε it appears there is no optimal value (Fig. 3).

We ran DBSCAN on the wind point dataset with *minPts* set to 2 and ε to 800 m, which yielded 23,534 clusters and 9,980 noise points (here representing single turbines or polygons intersecting no point data). We ran DBSCAN on the solar point dataset, again with *minPts* set to 2, and ε set to 400 m, which yielded 30,394 clusters and 4,878 noise points. 400 m was selected for solar as the mid-range value suggested by Fig. 3.

### Estimating power output of installations

The vast majority of variables (including capacity, rotor diameters, and areas) that would ordinarily provide a straightforward way of calculating power were >99% missing values in geospatial data from OSM. We overcome this limitation and provide first order power estimates derived solely from the area of the polygon and the number of original points contained within the OSM data, using regression equations derived from independent datasets.

The processed wind and solar OSM datasets were spatially joined with three independent datasets each. A spatial join combines the characteristics of any data that overlap each other in space. Using this technique, we can assign more descriptive metadata from national databases to the spatial information gleaned from the processed OSM data. For wind, these validation datasets were the USWTD, the United Kingdom Renewable Energy Planning Database (UK REPD), and data from a German renewable energy study^20–23^. Solar also utilised the German and UK data (these databases provide more than one type of renewable energy), swapping in data from Wiki Solar^26^ in place of the USWTD.

Spatial joins were performed using the *sf* package^34^. Duplicate matches, where more than one record spatially overlaps, were discarded in order to keep the model setup as simple as possible. This, for example, excluded instances where DBSCAN considered an area as one contiguous wind (or solar) farm and the corresponding national dataset considered the area as several different projects. This often happens when one larger installation is installed in successive funding rounds. Spatial joining with this caveat yielded 3096 instances for solar and 3457 for wind where we knew the OSM characteristics, but also other descriptors provided by the validation datasets, such as the power capacity.

**Fig. 4:**
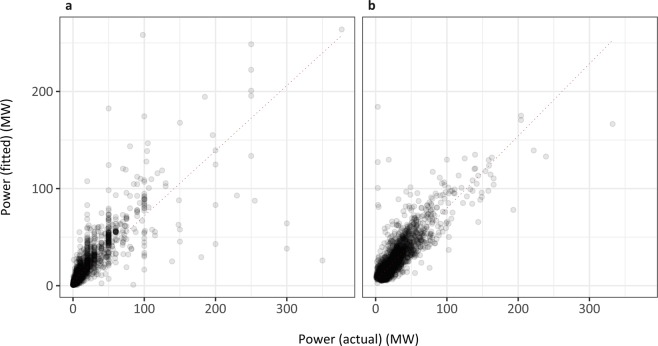
Fitted vs actuals for the solar (**a**) and wind (**b**) power models. Slope represents y~x, i.e. perfect prediction. 5-repeat 10-fold cross validation models: solar (RMSE = 14.29, *R*^2^ = 0.70, MAE = 4.93, *n* = 3280) and wind (RMSE = 11.12, *R*^2^ = 0.734, MAE = 6.22, *n* = 3574).

Two 5-repeat 10-fold cross validation models were trained on these data (Fig. 4) and used to predict power for the larger processed OSM solar and wind datasets. For solar, power was predicted from the installation panel area only, whereas for wind, power was predicted from both the number of turbines and the area of the installation. The power of a wind installation is dependent on the type of turbines installed; larger turbines require larger wake distances so are likely to be more sparsely spaced in an installation. Including landscape area as well as turbine number allowed us to create a *de facto* measure of turbine density. Predicting power solely from number of turbines implicitly assumes the same turbine type occurs at all global installations; predicting power from number of turbines only was inferior to the full model (RMSE 14.24434 > 11.23803).

The power estimates are the best available currently, but should be viewed with caution as, for example, the Wiki Solar dataset only records solar installations >100 MW. Furthermore, these datasets are from three early-adopting countries, where wind and solar projects are more established, engineering expertise is more readily available, and higher power projects are more likely. All three - USA, UK, and Germany - are in the top ten countries for solar and wind capacities in 2018. We understand that predicting power beyond the geographical range of the input data can be problematic. However, there is no reason to think that the relationship between solely geospatial predictors (area, number of points) and power would change with geography with the limited wind and solar technology currently available, and hence we feel that our extrapolation is reasonable and defensible given data limitations.

There were also 9 instances where the solar power model predicts power capacities in the larger OSM dataset beyond the power range present in the solar power model input data (where the maximum power capacity is 377 MW). However, the solar power model input data capture 99.9% of the variation in panel area in the larger OSM dataset, so we opted to include these 9 extrapolated points as a justifiable small extension of the model. The wind power model captured 100% of the variability in turbine numbers and 99.95% of the variation in landscape area in the larger OSM dataset, with no power capacities beyond the range found in the independent datasets.

## Data Records

**Fig. 5:**
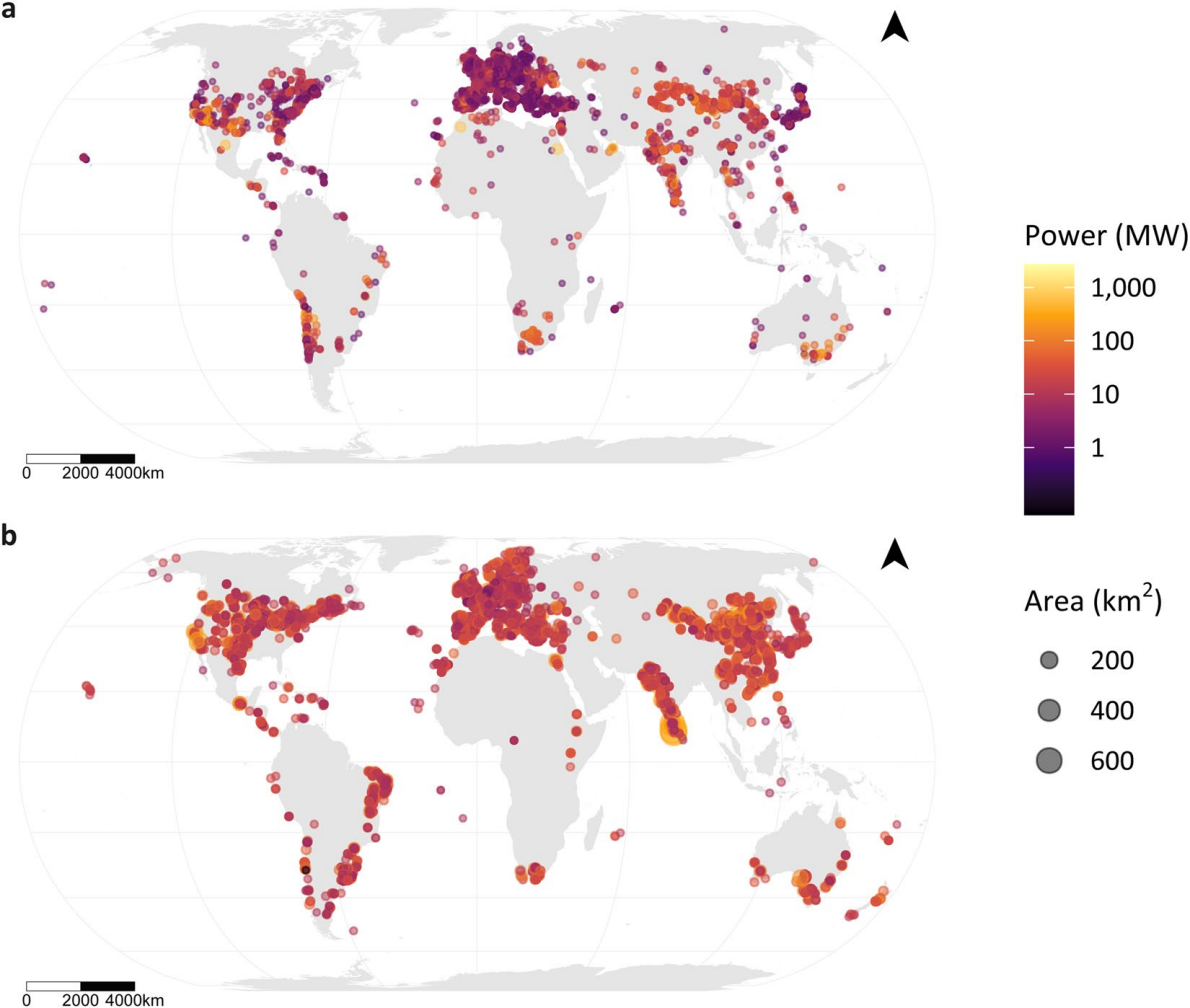
The global distribution of solar (**a**) and wind (**b**) installations. Solar installations represent those outside of urban cells and more than 1 hectare in panel area. Wind installations represent those outside of urban and water cells and with more than four turbines. ‘Area’ refers to landscape area for wind and panel area for solar.

This dataset is stored in three different formats: shapefiles for use with GIS software, geopackage for open-source usage, and .csv format for ease of use in any statistical software. Two final datasets were produced that represent the best publicly available global, harmonized geospatial data for field-scale solar PV and wind installations (Fig. 5). We provide vector data (point and polygon) for grouped installations (more than two features; Methods), in Eckert IV equal area projection.

### (1) Global solar PV installations

- global_solar_2020.gpkg
- global_solar_2020.csv
- global_solar_2020 layer in global_wind_solar_2020.gdb

### (2) Global wind installations

- global_wind_2020.gpkg
- global_wind_2020.csv
- global_wind_2020 layer in global_wind_solar_2020.gdb

Both datasets include the following variables:

- *x_id*; unique ID for data record
- *GID_0*; country ISO-3 code
- *panels* or *turbines*; the number of OSM tagged features that occurred within the boundaries of the cluster
- *panel.area* (solar only); the (estimated) area of panels in the cluster in km^2^
- *landscape.area*; the area of the site in km^2^, i.e. the area bounded by the outermost points or polygons, buffered by 800 m for wind
- *water*; binary indicator of whether the feature occurs in an area classified as water (Methods)
- *urban*; binary indicator of whether the feature occurs on land classified as urban (Methods)
- *power*; estimated power capacity in MW

The .csv and .shp files, in lieu of detailed spatial information, contain the X and Y coordinates of the data centroid. The geopackage format can contain multiple geometries and is the preferred option. To comply with ESRI field name specifications, landscape area and panel area are both renamed as *p_area* and *l_area* in the ESRI geodatabase.

The final, downloadable format of both databases is available from *Figshare*^35^.

## Technical Validation

### Data completeness assessment

To assess whether the OSM data truly reflect global solar (Fig. 1a) and wind built infrastructure (Fig. 1b), or simply sampling bias (most observations are in developed countries with large OSM user communities^36^), we ran regressions for the raw number of solar and wind features extracted from OSM per country as explained by their respective reported solar PV and onshore wind capacities per country in 2018, as well as other variables known to influence the completeness of OSM data.

While reported onshore wind capacity alone explains the number of wind feature observations relatively well (*R*^2^ = 0.90), the relationship between solar capacity and number of observations is a lot weaker (*R*^*2*^ = 0.13). However, removing data for China improves the solar correlation considerably (*R*^2^ = 0.52) due to an apparent dearth of OSM data compared to the enormous reported capacity.

One previous study assessing the OSM global road network identified three significant factors driving the completeness of a country’s data: land area, the number of Internet users, and the country governance^36^. We used the same World Bank variables from that analysis for the latter two: Internet users per 100 people in 2015, and the Voice and Accountability Governance indicator for 2018. This allowed us to assess whether the geographic variability in OSM features is driven primarily by the existence of wind and solar infrastructure (using reported capacity as a proxy), or factors relating to the completeness of OSM.

As number of observations represents count data, we looked at fitting Poisson general linear models. However, the number of observations per country for both solar and wind were heavily zero-inflated; i.e. the majority of countries worldwide do not have any renewable-tagged OSM data. Log transforming count data to correct for zero-inflation has previously been shown to have little use over alternative methods^37,38^, thus we decided to fit a zero-inflated negative binomial (ZINB) regression model. A zero-inflated negative binomial regression model was selected over the similar hurdle approach as we expect two types of zeroes in the data: *structural* zeroes, where a country with no renewable energy capacity could never have any renewable infrastructure, and *sampling* zeroes, where a country may have national renewable energy capacity, but no data in OSM. A ZINB first fits a binomial regression to the data to produce an estimate of the count being positive, then fits a truncated negative binomial model to produce an estimate of the count. The predictors do not have to be the same in both models.

For our application, the models fitted solely with reported capacity outperformed the models with only the three OSM completeness metrics (Likelihood ratio test, p < 2.2e^−16^ and p = 0.001659 for wind and solar respectively). However, the most parsimonious models are presented in Tables 3 and 4 below, and these include some measures of OSM data completeness. National capacities and land area were both modified with a Yeo Johnson transformation to correct for heavy negative skew. All variables were scaled.

**Table 3:** Zero-inflated negative binomial regression fitted for OSM wind observations (*n* = 128). Whether a country has any wind observations at all is driven by the reported wind capacity, after which the capacity, governance and land area of the country explain the count.

Coefficient	Estimate	Std. Error	z value	Pr(>|z|)	Odds ratio
**Count model coefficients (negbin with log link)**					
(Intercept)	−0.6220	0.3173	−1.960	0.04994*	0.5369
Wind capacity 2018	2.6145	0.1236	21.158	<2e−16***	13.6608
Governance 2018	0.2703	0.1047	2.582	0.00982**	1.3103
Land area	0.3547	0.1224	2.899	0.00375**	1.4258
Log(theta)	0.5579	0.1500	3.718	0.00020***	
**Zero-inflation model coefficients (binomial with logit link)**					
(Intercept)	0.3986	0.5336	0.747	0.455120	1.4897
Wind capacity 2018	−2.5573	0.6945	−3.682	0.000231***	0.07752
Theta = 1.747					
Log-likelihood: −668.6 on 7 Df

**Table 4:** Zero-inflated negative binomial regression fitted for OSM solar observations (*n* = 178). Whether a country has any solar observations at all is driven by the reported solar capacity, after which the capacity, governance and land area of the country explain the count.

Coefficient	Estimate	Std. Error	z value	Pr( > |z|)	Odds ratio
**Count model coefficients (negbin with log link)**					
(Intercept)	0.9262	0.4371	2.119	0.0341*	2.5250
Solar capacity 2018	1.4065	0.1775	7.922	<2.33e-15***	4.0816
Governance 2018	1.1329	0.1909	5.933	2.97e-09***	3.1048
Land area	0.8625	0.1942	4.440	8.98e-06***	2.3690
Log(theta)	−1.1101	0.1214	−9.144	<2e-16***	
**Zero-inflation model coefficients (binomial with logit link)**					
(Intercept)	−0.2662	0.7886	−0.338	0.7357	0.7663
Solar capacity 2018	−1.7696	0.7805	−2.267	0.0234*	0.1704
Theta = 0.3295					
Log-likelihood: −1120 on 7 Df					

Although country governance and land area contribute towards the variability in OSM observations for both wind and solar, the odds ratios for the national capacities clearly suggest that the observed pattern is largely reflective of the true distribution of renewable infrastructure. Furthermore, the sole driver of whether a country has any OSM data at all, e.g. the binomial models, in both cases, is the reported national capacity.

Moreover, after data processing, the aggregated estimated power capacities of our datasets per country correlate even better with reported capacities (*R*^2^ = 0.82 and 0.97 for solar and wind respectively).

### DBSCAN *minPts* parameter

**Fig. 6:**
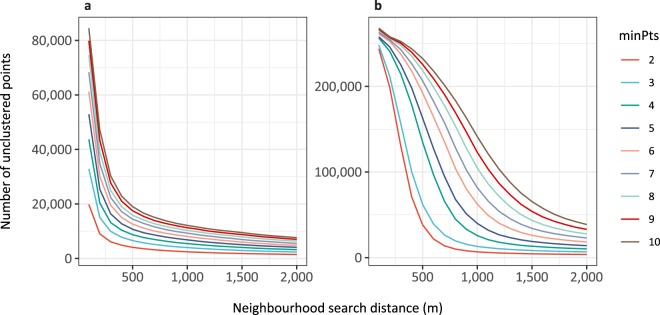
“Knee” plots for OSM solar (**a**) and wind (**b**) point data showing the number of unclustered points remaining for differing neighbourhood search radii and values of *minPts*. For wind, the optimal value of ε changes as minPts increases, but the value for solar remains relatively constant.

To assess the functioning of DBSCAN when passed a *minPts* argument of 2 over more explicit density-based clustering, and to check whether the model fits were adversely affected by the *de facto* single-linkage clustering needed to identify some linear wind installations, we repeated the power analyses for different values of *minPts* and ε. Figure 6 shows the ‘knee’ plots for solar (a) and wind (b). For solar, the value of *minPts* does not appear to affect the appropriate value for the neighbourhood search radius (ε) of 400 m. The optimal neighbourhood search radius does appear to change with *minPts* for wind. The values selected for ε are shown in Tables 5 and 6.

**Table 5:** Performance measures for 5-repeat 10-fold cross validation models fitted to predict the power capacity of wind installations. Here, *n* is the sample size for the spatial join with independent datasets (Methods) after processing the raw OSM data with the presented values of *minPts* and ε.

minPts parameter	ε (m)	RMSE	*R*^2^	MAE	*n*
2	800	11.22133	0.7308343	6.233574	3574
3	800	10.80424	0.7516774	6.134675	3571
5	1000	11.68232	0.7308547	6.776778	2678
10	2000	19.71299	0.6271118	11.54724	848

**Table 6:** Performance measures for 5-repeat 10-fold cross validation models fitted to predict the power capacity of solar installations. Here, *n* is the sample size for the spatial join with independent datasets (Methods) after processing the raw OSM data with the presented values of *minPts* and ε.

minPts parameter	ε (m)	RMSE	*R*^2^	MAE	*n*
2	400	18.42949	0.6482381	5.367391	3280
3	400	17.90751	0.6519353	5.263504	3396
5	400	17.60908	0.6473003	5.254524	3558
10	400	17.55904	0.6446935	5.211948	3621

As the primary purpose of our fitted power models is prediction, and not explanation, the root mean square error (RMSE) and mean absolute error (MAE) are the most appropriate performance measures for these models. The RMSE and MAE of the wind models are highest as the *minPts* parameter is increased to 10 (Table 5), but notably the models trained on *minPts* 2, 3 and 5 are very similar, suggesting that the wind data are not significantly affected by the lack of specifically *density*-based clustering. The solar data appear to be robust against different values of DBSCAN parameters (Table 6).

## Usage notes

Location data for wind and solar installations worldwide can be used to support a range of applications, including analysing the land impact of current infrastructure, measuring progress towards global goals, and informing future energy planning scenarios.

Ongoing work involves the integration of these datasets with socioeconomic and biophysical predictors to produce probability surfaces for the likely development of wind and solar infrastructure in order to more accurately highlight potential trade-offs with other important sustainable land uses^15^. While potential regions of conflict have been highlighted in previous studies, for example biodiversity and renewable energy^8,9^, these new data allow analysis at whatever resolution there are readily accessible global predictors (currently 1 by 1 km grids). This is especially important as it has been suggested that the wider social and environmental impacts of energy scenarios are typically overlooked because the majority of scenarios are aspatial^39^.

Additionally, there are many applications of these data outside a purely renewable energy context. While this analysis focuses on renewable energy infrastructure, there is no reason why the methodology cannot be replicated for other types of infrastructure lacking in openly accessible data. For example, conventional fuels also lack such consistent data. Appropriate tags for oil and gas can be found at the site of the Oil and Gas Infrastructure WikiProject^40^. We would caution that the specific methodology of this study was designed with renewable energy in mind, and some thought would be needed to recalibrate some of the analysis parameters, e.g. the neighbourhood distance.

R scripts are provided that allows users to generate and process their own raw OSM data at a future date. All model data is also provided so that users can recreate the power models and compare to reported national capacities.

We highly recommend using the geopackage data, which can be easily read into R with the *sf* package and has the advantage of holding multiple geometries, i.e. point, multipoint, polygon and multipolygon data. For ease, the shapefiles and comma-delimited files were restricted to point geometries by taking the centroid of each data record.

The datasets contain all data and require filtering in order to be meaningful for different use cases. For example, the power models in this paper were trained on a subset of the raw data: solar farms not in urban centres, and with a panel area of >1 ha, and wind farms not in urban centres or in water and with more than four turbines (the median number of turbines in the three independent datasets was 5). For this reason, power capacity is missing for all data that do not meet these criteria.

## Data Availability

The code used to extract and process the OSM data is publicly available through the *Figshare* repository^35^. The code consists of four R programming language scripts (R version 3.6.2) numbered 1-4: the first extracts the latest OSM data; the second processes the data into wind and solar farms; the third contains the power models, and the fourth conducts the technical validation. Each script includes text that guides the user through the process and details the functions being performed. The README file, included with the scripts, provides more detail on rerunning the analyses. We regret that we cannot provide the full, geospatial Wiki Solar dataset as it was provided on the condition of confidentiality. We have provided a copy of the spatial join between the Wiki Solar dataset and the processed OSM data, with all geospatial data stripped out. This can be used as an input to the power estimations on its own. However, the power estimation can be rerun omitting these data if users require models trained on truly open-access data. Alternatively, users can contact the Wiki Solar data provider. When we ran this analysis, the accuracy of the solar model to predict unseen data in the two remaining independent datasets increased (RMSE = 3.153742, *R*^2^ = 0.7442277, MAE = 1.386226, *n* = 1889). However, the input data for this model only managed to capture 96.8% of the variation in panel area in the wider OSM dataset and subsequently predicted 253 occurrences of power capacities outside of the model range. For this reason, we elected to keep the Wiki Solar data in the final model.
